# The role of environmental factors to transmission of SARS-CoV-2 (COVID-19)

**DOI:** 10.1186/s13568-020-01028-0

**Published:** 2020-05-15

**Authors:** Hadi Eslami, Mahrokh Jalili

**Affiliations:** 1 Occupational Environment Research Center, Department of Environmental Health Engineering, School of Health, Rafsanjan University of Medical Sceiences, Rafsanjan, Iran; 2 Environmental Science and Technology Research Center, Department of Environmental Health Engineering, School of Public Health, Shahid Sadoughi University of Medical Sciences, Pardis Campus, Gomnam Blv, Alem Squre, Yazd, Iran

**Keywords:** Coronavirus, COVID-19, Environmental factors, Inanimate surfaces

## Abstract

The current outbreak of the novel coronavirus disease 2019 (COVID-19) in more than 250 countries has become a serious threat to the health of people around the world. Human-to-human transmission of the Severe acute respiratory syndrome coronavirus 2 (SARS-CoV-2) occurs most often when people are in the incubation stage of the disease or are carriers and have no symptoms. Therefore, in this study, was discussed the role of environmental factors and conditions such as temperature, humidity, wind speed as well as food, water and sewage, air, insects, inanimate surfaces, and hands in COVID-19 transmission. The results of studies on the stability of the SARS-CoV-2 on different levels showed that the resistance of this virus on smooth surfaces was higher than others. Temperature increase and sunlight can facilitate the destruction of SARS-COV-2 and the stability of it on surfaces. When the minimum ambient air temperature increases by 1 °C, the cumulative number of cases decreases by 0.86%. According to the latest evidence, the presence of coronavirus in the sewer has been confirmed, but there is no evidence that it is transmitted through sewage or contaminated drinking water. Also, SARS-COV-2 transmission through food, food packages, and food handlers has not been identified as a risk factor for the disease. According to the latest studies, the possibility of transmitting SARS-COV-2 bioaerosol through the air has been reported in the internal environment of ophthalmology. The results additionally show that infectious bio-aerosols can move up to 6 feet. There have been no reports of SARS-COV-2 transmission by blood-feeding arthropods such as mosquitoes.

## Introduction

**Fig. 1:**
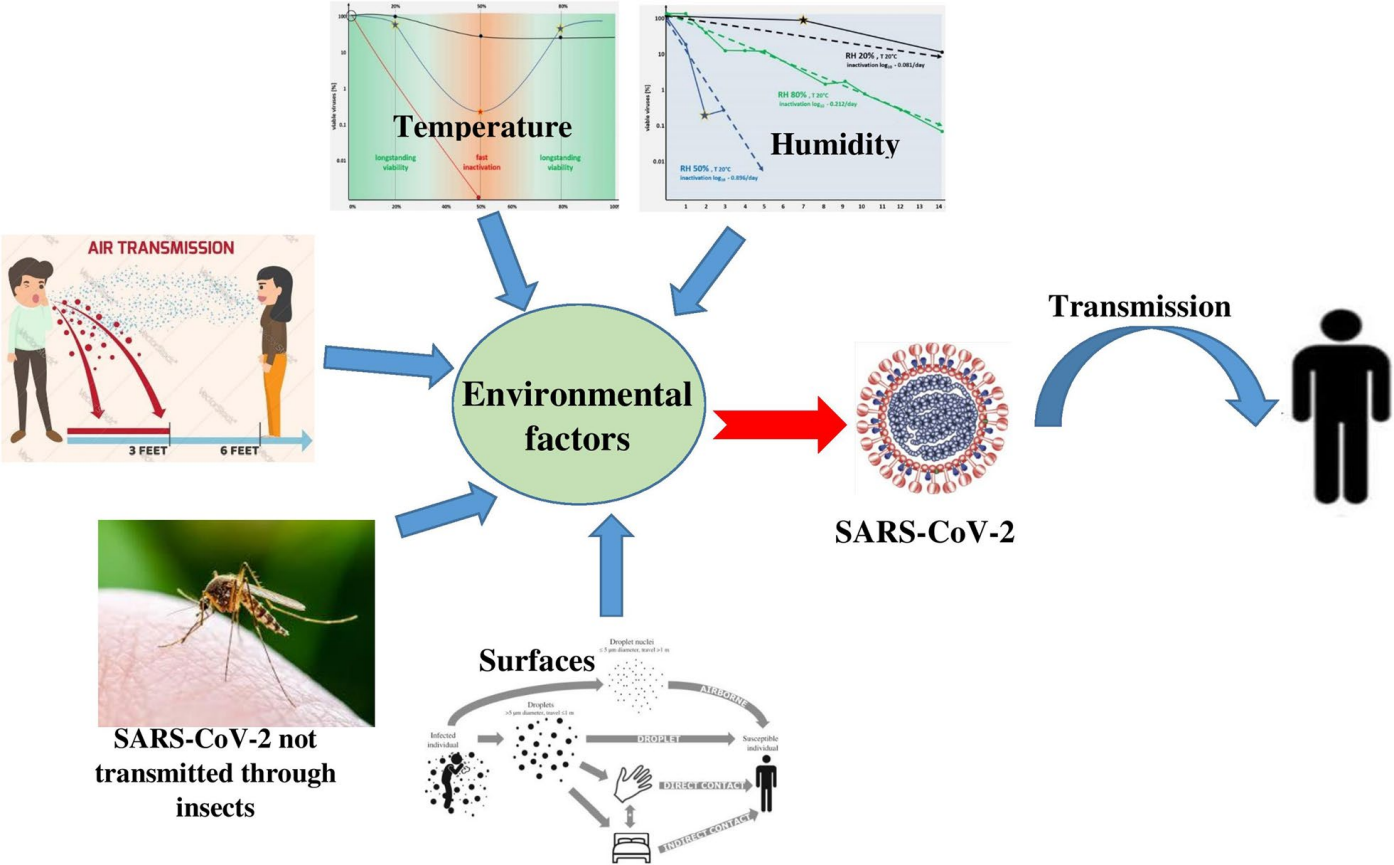
Environmental factors and transmission of SARS-CoV-2

The new outbreak of COVID-19 has recently become a serious threat to the health of people around the world. COVID-19 is caused by the SARS-CoV-2, which is a single-stranded positive-sense RNA virus that causes infection and respiratory failure (Swerdlow and Finelli 2020; Wu et al. 2020) and has led to the thousands of people deaths (Thienemann et al. 2020). Following the WHO announcement, the Global Emergency Committee identified the need for early detection, quarantine, and prompt treatment as a global concern (Sohrabi et al. 2020), because people with the virus do not have clinical symptoms such as fever, cough, etc., in the early stages. For this reason, there is not enough information on how to disinfect and disinfect surfaces and hands, human-to-human transmission through air, food, and water, and the presence of the virus in human wastewater and waste. There are several factors involved in transmitting the virus. These conditions can be included in environment and human behavior (Fig. 1). The distribution of human population, migration, social interactions, climate change (deforestation, habitat invasion), agricultural growth, and direct contact with domestic and wild animals fall into this category (Barratt et al. 2019; Dehghani and Kassiri 2020). On the other hand, the effect of environmental factors such as ambient temperature, humidity, etc., relating to the COVID-19 pandemic (Fig. 1) has not been sufficiently investigated. How the virus enters the body (eyes, ears, mouth, and nose) is not well known by the release of aerosols and droplets containing SARS-CoV-2 in human societies. However, previous findings in the epidemic of a virus similar to SARS-CoV-2 can be used in this regard (Sun et al. 2020), because the methods of transmission are not currently known. Therefore CDC recommends standard precautions, contact and air, and eye protection (glasses or face shields) (CDC 2020b, e). It has been reported that even with personal protective equipment (PPE), it is still possible to get infected (Dai 2020) because there is no convincing evidence to support the effect of PPE use in the prevention of infection (Yang 2020). The significant concern about SARS-CoV-2 is its transmission (Dehghani and Kassiri 2020). According to the WHO, there is no certainty about the persistence of the SARS-CoV-2 on surfaces. However, the virus appears to act like other coronaviruses and can survive on the surface for at least several hours (Parry 2004). For this reason, the following health points in public places, hospitals, and residential houses has a significant role in controlling the transmission of the disease (WHO 2020a). Dealing with COVID-19 and preventing its rapid and dangerous spread is a global challenge. Therefore, the fight against this disease requires global management. However, due to the potential variability of this disease, according to the type of climate and other environmental factors, its prevention and control should be investigated quickly and seriously (Askari et al. 2018). When a new pandemic occurs, a lot of health advice is given. Anyway, it should be noted that these methods may not be the most accurate and correct, but following them can be effective until sufficient knowledge is obtained. Nevertheless, a hierarchy to deal with SARS-CoV-2 transmission involves three steps: 1) self-care, 2) control of environmental factors, and 3) the use of PPE (Lai et al. 2020). This study aimed to investigate the effect and role of various factors, including environmental factors (climate change, water transfer, air, and food), disinfection of surfaces, and hands in the transmission and prevalence of COVID-19 pandemics.

## Environmental condition and transmission of SARS-CoV-2

Currently, due to the prevalence of COVID-19 in most parts of the world, one of the primary concerns is the relationship between environmental factors such as rising summer temperatures and the rapid prevalence of coronavirus (Abbasi et al. 2020; Wang et al. 2020b). One study observed the relationship between the numbers of positive daily SARS-CoV-2 cases with three environmental factors: maximum relative humidity, maximum temperature, and maximum wind speed in four cities in China and five cities in Italy. In this study, the relationship between the prevalence of the COVID-19 with maximum air humidity and wind speed was negligible and statistically not significant. Although, in most cases, with increasing humidity and wind speed, the prevalence has decreased. The association between COVID-19 prevalence and maximum ambient temperature was negligible to moderate. Also, with increasing temperature in most of the studied cities, the prevalence of the disease has decreased (Bhattacharjee 2020). The study by Chin et al. (2020) reported the resistance of the SARS-CoV-2 at 4 °C for a long time, but at 70 °C, its resistance was 5 min. In general, heat, high or low pH, and sunlight make it easier to kill the coronavirus (WHO 2020b). However, the results of a study also showed that the virus is stable at different pHs of 3–10, at room temperature (Chin et al. 2020). A new study of 24,139 positive SARS-CoV-2 cases was conducted in 26 regions in China. Its results show that with a 1 °C increase of the minimum ambient air temperature, the cumulative number of cases decreases by 0.86% (Fig. 1) (Wang et al. 2020b).

## Coronavirus transmission by Food, food packages, and food handlers

According to the CDC, SARS-CoV-2 transmission through food, food packages, and food handlers has not been identified as a risk factor for the disease. However, based on the available evidence and the persistence of the virus on the surfaces between a few hours and a few days, CDC suggested washing and then disinfection as the best way to control this virus (Seymour et al. 2020b). Sanitary recommendations are also recommended when relocation and preparing food, such as washing hands regularly, separating raw meat from other foods, cooking at high temperatures, and storing food in the refrigerator (Eslami et al. 2015; Seymour et al. 2020c). The most important health recommendations for staff involved in preparing and distributing food include complete personal hygiene such as covering the nose and mouth by bending the elbow during sneezing or coughing, isolating employees with COVID-19 symptoms or having contact with these patients, and following the minimum distance, 6 feet or 1.8 m, between employees. Also, providing multiple points for hand washing and disinfection at work and washing and disinfection of surfaces with high contact by diluted sodium hypochlorite (0.1%) (Seymour et al. 2020c).

## Water and wastewater and transmission of COVID-19

Guaranteeing safe water, collecting sewage, and maintaining effective hygiene during infectious diseases, including COVID-19 pandemic, play a key role in supporting human health (Eslami et al. 2018; WHO 2020b). The risk of COVID-19 appears to be low through the stool of an infected person. There is evidence that SARS-CoV-2 may lead to intestinal infection and be present in the stool (Bhattacharjee 2020). Studies show that approximately 2 to 10 percent of confirmed SARS-CoV-2 cases have been associated with diarrhea (Chen et al. 2020; Huang et al. 2020; Wang et al. 2020a). Two studies have reported the detection of residual SARS-CoV-2 viral RNA in the stool of patients with SARS-CoV-2 (Holshue et al. 2019; Xiao et al. 2020). Yet, only one study has reported the SARS-CoV-2 from a cultured stool sample (WHO 2020b). In the latest study, the report of the presence of the SARS-CoV-2 virus in sewage has been confirmed. The presence of the virus in the wastewater, even when the prevalence of the virus is low, indicates that the SARS-CoV-2 can survive in the sewage. This finding could be a sensitive tool for tracking and monitoring the rate of virus rotation in communities (Lodder and de Roda Husman 2020; Medema et al. 2020). Some studies have reported that coronavirus can remain in water or wastewater sources for days or weeks (Qu et al. 2020). Due to the evidence of the virus surviving in aquatic and sewage environments, its presence in water resources is dependent on essential factors such as temperature, sunlight, and the presence of organic compounds that the virus can adsorb to and protect themself against sunlight. The presence of other antagonistic microorganisms can also affect its survival in water resources. According to the latest WHO report, there is no evidence that human-type coronavirus has been transmitted through contaminated drinking water (Naddeo and Liu 2020). In general, coated viruses are less environmentally friendly and are more sensitive to oxidants such as chlorine. The SARS-CoV-2 is likely to be significantly inactivated more rapidly than human intestinal viruses without water-borne diseases in contact with oxidants (WHO 2020b).

## Air and transmission of COVID-19

Research has shown that newly infected patients have no clinical symptoms and can easily be the source of the spread and transmission of SARS-CoV-2. This transmission can be through sneezing, coughing, talking to other people at close range, and through the foul airflow (Li et al. 2020; Zhi 2020). Based on the available evidence, it is not thought that air diffusion is one of the main transmission routes (Dehghani and Kassiri 2020). Non-invasive ventilation, high flow oxygen therapy, intubation/extubation, nebulization, open suctioning of airway secretions, bronchoscopy, induction of sputum, bag and mask ventilation, cardiopulmonary resuscitation, etc. are the principal causes of SARS-CoV-2 aerosol release in airflow (CDC 2020d). For example, the role of environmental pollution in several hospitals has been evaluated following the outbreak of MERS-CoV in 2015 in Korea, as well as experimental studies on the survival and stability of MERS-CoV on surfaces and air (Bin et al. 2016). There is also the possibility of air stability for SARS-CoV-2. Therefore, the use of viral filters and closed suctioning, airflow changes, and negative pressure air pollution in closed environments, with the probability of infection, is recommended (Malhotra et al. 2020). Thus, people in contaminated environments should use a mask and, if there is no mask, should use face masks (Bowdle and Munoz-Price 2020). Facial hair can provide a way for SARS-CoV-2, to penetrate (Malhotra et al. 2020). Using non-standard masks can also exacerbate the incidence of SARS-CoV-2 (Jones and Xia 2018). Besides, wearing a mask in some health workers has shown symptoms of claustrophobia, respiratory distress, discomfort, and skin irritation (Barratt et al. 2019). The most available type is the N95 mask, which according to studies can be sterilized and reused by hydrogen peroxide, gamma rays from CO60, UV rays, dry and wet heat, and ethylene oxide. In the meantime, the use of radiation is preferable to other methods (Card et al. 2020; Cramer et al. 2020; Kenney et al. 2020). Gamma CO60 with a dose of 10 KG can sterilize the N95 mask used in SARS-COV environments, and kill the virus (Feldmann et al. 2019). However, high doses of radiation may damage the fibers of the mask and allow air to pass through without purification, but it does not have secondary radiation and its side effects (Harrell et al. 2018). Hydrogen peroxide is also a useful antiviral and has no adverse effects on the respiratory tract (Viscusi et al. 2009). Enhanced Traffic Control Bundling (eTCB) was very effective at the time of the SARS-COV outbreak. Due to the similarity of this disease with COVID-19, the application of this strategy can prevent the transmission of the disease through the air. (Yen et al. 2020). In this way, patients with clinical symptoms quarantined, other people use disinfectant and PPE during patients’ visits to the hospital. This process has been approved in the control of other epidemic diseases such as Ebola and SARS-CoV and is currently being implemented in COVID-19 pandemics (Ong et al. 2020, Schwartz et al. 2020b; Yen et al. 2015). The possibility of transmitting bioaerosols with the ability to move up to 6 feet through the air has also been reported in the internal environment of ophthalmologists (CDC 2020c). As a result, HEPA filters have been used for ventilation. Still, air-born emissions for SARS-CoV-2 have not yet been reported. According to available evidence, it is not confirmed that air can be one of the principal causes of transmission. Therefore, it can be predicted that if any suspended matter is created in the air of medical centers, the transfer will be easy (WHO 2020a).

## Insects and transmission of SARS-CoV-2

Numerous factors are contributing to the spread of global diseases such as COVID-19, which is spreading worldwide. These conditions include ecology and human behavior. These cases have been directly and indirectly involved in the occurrence of some problems and diseases (Barratt et al. 2019; Dehghani and Kassiri 2020). Most coronaviruses are pathogenic and can cause acute respiratory syndrome. So far, 7 types of them have been identified (Gorbalenya 2020; Roush et al. 2019). The transmission and evolution of the SARS-CoV-2 from bats to scaly anteaters (pangolins) and then to humans has been reported (Dehghani and Kassiri 2020). This process is now present in humans through aerosols, but transmission through secretions such as stool has not been confirmed. In addition to these cases, the transfer through bandages can also be considered. There have been no reports of SARS-CoV-2 transmission by blood-borne arthropods such as mosquitoes (Shankar et al. 2020; Wu and McGoogan 2020). Some studies have associated insects such as beetles and domestic insects, which are the main mechanical carriers of pathogens, by contact with contaminated surfaces and patients’ secretions involved in transmission (Graczyk et al. 2005; Kobayashi et al. 1999; Vazirianzadeh et al. 2014). SARS-CoV-2 excretion by stool has been confirmed in some patients. Therefore, feeding domestic insects and beetles from the stool and its mechanical transmission can play a significant role in the transmission of the disease (Parry 2004). They can transmit more than 100 pathogens through the legs, body hair, mouth, feces, and vomit. In this regard, the elimination of mechanical carriers is very important not only in residential homes but also in public places. There are several ways to control these organisms, including improving environmental hygiene, such as putting garbage in closed bags and trash cans, controlling sanitary landfills, sanitary toilets, proper sewage disposal systems, and preventing the accumulation of manure nearby, and residential areas (Jobara 2016). Clinical and laboratory wastes from SARS-CoV-2 detection in suspected individuals, secretions such as blood (1%) and feces (26%) can also be a major source of the virus (Chang et al. 2020; Iwen et al. 2020). But no study has shown the presence of the virus in the urine (Wang et al. 2020c). Finally, if such waste is not properly managed, SARS-CoV-2 can be transmitted through mechanical transmission by insects (CDC 2020a).

## Inanimate surface and disinfection

Human-to-human transmission of the SARS-CoV-2 occurs most often when people are in the communal stage of the disease or carrier and have no symptoms, or when they are with mild symptoms. The important ways of transmitting the virus are through Droplets, infected hands, and skin-to-skin contact, as well as inanimate surface contact (Kampf et al. 2020). Studies show that SARS-CoV-2 stays for more than 9 days and be resistant, like other coronaviruses such as SARS and Middle East Respiratory Syndrome (MERS), can live on inanimate surfaces such as metals, glass, and plastic at room temperature. Also, at ambient temperatures of 30 °C and above, the duration of SARS-CoV-2 is reduced (Kampf et al. 2020). Some studies have suggested that a temperature of 56 OC for 90 min or a temperature of 67 OC for 60 min can cause SARS-CoV-2 to be inactive (Henwood 2020). Kampf et al. (2020) showed that at room temperature and 50% humidity, the resistance of the virus was more than 30%. In one study, the stability of SARS-CoV-2 on different surfaces confirmed that the virus was more resistant to smooth surfaces than others. According to the results of this study, the virus remains stable at temperatures of 22 oC and relative humidity of 65% for 4 days on surfaces such as glass and banknotes and 7 days on smooth surfaces such as steel and plastic. It also stays on rough surfaces such as fabric and wood for up to 2 days (Chin et al. 2020). Another investigation, which examined environmental levels of SARS-CoV-2 virus in hospital centers, found that the most important environmental levels of infection were self-service printers (20%) (used in Chinese laboratories), desktops, and keyboards (16.8%), door handles (16%), telephones (12.5%), and medical equipment (12.5%) (Ye et al. 2020). Studies show that students touch their face with their hands an average of 23 times an hour. 56% of it is the touch of the skin, 36% is the lips, 31% is the nose, and 31% is the eyes (Kwok et al. 2015). Although SARS-CoV-2 is not available on the surface of complete information on viral load, it can be said that by reducing the frequency of touching surfaces by hands and disinfecting surfaces, the amount of virus load on surfaces can be reduced (Kampf et al. 2020). The WHO has recommended that cleanliness be done with water and detergents to ensure environmental cleanliness, and then the use of common environmental disinfectants, such as sodium hypochlorite (WHO 2020c). The most well-known methods of surface disinfection to remove SARS-CoV-2 virus are, in short, the use of ethyl alcohol (62–70%), or hydrogen peroxide (0.5%) or sodium hypochlorite (0.1%, dilution ratio 1 to 50) with a contact time of 1 min (Henwood 2020; WHO 2014). Other biocidal agents such as benzalkonium chloride (0.05–0.2%) or chlorhexidine digluconate (0.2%) are also applicable, but their effectiveness is less than previous (WHO 2014). The effect of hand soap on disinfecting surfaces from the SARS-CoV-2 virus was investigated. The results of this study show that the virus is inactivated by contact with soap for 5 min (Chin et al. 2020). The CDC has also recommended a water temperature above 65 degrees to disinfect surfaces (Seymour et al. 2020a). One of the most important concerns at the pandemic period of coronavirus is disinfection and sterilization of surgical masks and N95 for reuse. The significant thing is that these devices are disposable and cannot be reused in normal conditions. However, in cases of shortage and under certain conditions, they can be reused up to 5 times by disinfecting and sterilizing them. The use of thermal methods of at least 50 °C for at 30 min is highly recommended as a disinfection and sterilization method for n95 masks. In one study, the use of Uc radiation with a 100 µwcm^−2^ power with a contact time of 15 to 20 min for sterilization of N95 mask was suggested (Card et al. 2020). In another study, the use of hydrogen peroxide (HP) vapor was used in a short time to sterilize the N95 mask (Seymour et al. 2020a). Hydrogen peroxide gas is suitable for disinfecting devices used for patients with viral infections. It also does not hurt patients’ respiratory system and can be used to disinfect hospital rooms (Schwartz et al. 2020a).

## Hand and eye hygiene

Preliminary health advice when spreading epidemics is to observe hand hygiene and use PPE. Some of the reasons for not following the recommendations occur at the individual, group, and community levels. At the time of the epidemic, such issues are particularly well addressed among medical staff. Nevertheless, some hand disinfectants are not effective in eliminating the pathogen (Pittet 2001). In other words, a disinfectant should be used that will affect the pathogen and the virus in question. At the same time as the COVID-19 pandemic, the effectiveness of hand sanitizers can prevent the virus from spreading, because the main way to prevent the transmission of pathogens is body contact. Lack of public access to such materials is also a significant issue at this time. The effectiveness of home disinfectants has been investigated by WHO (Seymour et al. 2020d). Its main ingredients are alcohol, glycerin, and hydrogen peroxide. After that, spray on the hands and drying is done automatically by exposing it to the air. Lack of hand disinfection and contact with parts of the body (ears, eyes, nose, and mouth) can cause COVID-19. In one study, alcohol-based hand rubs, or soap and water hand wash, were shown to be effective in preventing COVID-19. Sequentially, if the disinfectant dries, the above-mentioned items should be used again before contact with the patient or any use of the hand (Malhotra et al. 2020). Although there is evidence that hand hygiene can reduce respiratory illness, it has not yet been proven that it can reduce the transmission of SARS-CoV-2 (Yang 2020). So far, no eye infections have been reported in patients with the SARS-CoV-2, but very few viruses have been seen in tears and conjunctival discharge. It can be seen that the eye may not be the main entrance of SARS-CoV-2 into the body and cause respiratory infections (Sun et al. 2020). However, eye infections can be caused by respiratory and nasopharyngeal infections. It can be very efficient, especially during close contact between the patient and the clinical staff. The use of masks and goggles, along with hand disinfection, has shown a significant reduction in person-to-person transmission (Yang 2020). Also, since SARS-CoV-2 is not only transmitted through the air and can survive on the surface for a while, the importance of hand disinfection cannot be ignored (Otter et al. 2016). Besides, infected people can contaminate the surface they touch, which may include a large number of household items and appliances (WHO 2020a). In this way, even infected people should disinfect their hands.

## Conclusion

Reviews of studies have shown that reducing the frequency of touching surfaces by hands and disinfecting surfaces can reduce the amount of coronavirus load on surfaces and the rate of transmission. The most important solution is to disinfect surfaces, clean surfaces with water and detergents, and then disinfect with ethyl alcohol (62–71%), or hydrogen peroxide (0.5%) or sodium hypochlorite (0.1%) with 1 min contact time. Despite the lack of evidence of SARS-CoV-2 transmission by blood-borne arthropods such as mosquitoes, some studies have identified insects such as beetles and domestic insects, which are the main mechanical carriers of pathogens, through contact with contaminated surfaces and patients’ secretions. Also, its transmission through the air with a minimum distance of 6 feet, water and sewage, and food has not been provided so far.

## Data Availability

Not applicable.
